# Neurofilament Isoform Promoter-Driven Expression of Therapeutic Genes

**DOI:** 10.1167/tvst.13.9.30

**Published:** 2024-09-30

**Authors:** Axel Petzold

**Affiliations:** 1 Moorfields Eye Hospital & UCL, London, UK.; 2 Department of Ophthalmology, Amsterdam UMC, Amsterdam, Netherlands. e-mail: a.petzold@ucl.ac.uk

I am writing to commend the team from the University of Pennsylvania on their innovative approach of selecting the neurofilament heavy chain promoter for optimizing the packaging of therapeutic genes in a viral vector strategy.^1^ It is a thoughtful and logical choice to target promoters of proteins expressed in neurons for neuroprotective genetic therapies, particularly when relying on vitreal injection, as it increases the likelihood that only retinal neurons will express the transferred genes.

In their conclusion, the authors note that gene expression efficacy was not superior for the neurofilament heavy chain promoter compared to conventional promoters. However, I would like to suggest a potential variation of this approach that could be explored in future research to enhance the expression logistics of neuroprotective retinal gene therapies.

The neurofilament heavy chain is part of a family of five neurofilament isoforms that are obligate heteropolymers.^2^ Research over the past two decades has consistently shown that, rather than the neurofilament heavy chain, it is the neurofilament light chain whose expression increases with neurodegeneration.^3^ This can be attributed to the reduced time and energy (adenosine triphosphate) required for the translation of the neurofilament light chain (543 amino acids) compared to the larger neurofilament heavy chain (1020 amino acids).^4^

Therefore, it would be highly valuable to compare the efficacy of neurotherapeutic gene expression using promoters from all five neurofilament isoforms in both acute^5^ and chronic^1^ retinal neurodegeneration models, as well as in control conditions. Such an investigation could provide new insights into optimizing gene therapy strategies for neurodegenerative diseases.
